# Factorial structure of the locomotor disability scale in a sample of adults with mobility impairments in Bangladesh

**DOI:** 10.1186/s12955-018-0903-1

**Published:** 2018-05-02

**Authors:** Ilias Mahmud, Lynda Clarke, Nazmun Nahar, George B. Ploubidis

**Affiliations:** 1 BRAC James P Grant School of Public Health, BRAC University, 68 Shahid Tajuddin Ahmed Sharani, Mohakhali, Dhaka, 1212 Bangladesh; 2 London School of Hygiene and Tropical Medicine, Keppel Street, London, WC1E 7HT UK; 3 Bangladesh Health Professions Institute, Centre for the Rehabilitation of the Paralysed, CRP-Chapain, Savar, Dhaka, 1343 Bangladesh; 4 Centre for Longitudinal Studies, Department of Social Science, University College London, 55 – 59 Gordon Square, London, WC1H 0NU UK

**Keywords:** Disability, Locomotor disability, Physical disability, Locomotor disability scale, Bangladesh

## Abstract

**Background:**

Disability does not only depend on individuals’ health conditions but also the contextual factors in which individuals live. Therefore, disability measurement scales need to be developed or adapted to the context. Bangladesh lacks any locally developed or validated scales to measure disabilities in adults with mobility impairment. We developed a new Locomotor Disability Scale (LDS) in a previous qualitative study. The present study developed a shorter version of the scale and explored its factorial structure.

**Methods:**

We administered the LDS to 316 adults with mobility impairments, selected from outpatient and community-based settings of a rehabilitation centre in Bangladesh. We did exploratory factor analysis (EFA) to determine a shorter version of the LDS and explore its factorial structure.

**Results:**

We retained 19 items from the original LDS following evaluation of response rate, floor/ceiling effects, inter-item correlations, and factor loadings in EFA. The Eigenvalues greater than one rule and the Scree test suggested a two-factor model of measuring locomotor disability (LD) in adults with mobility impairment. These two factors are ‘mobility activity limitations’ and ‘functional activity limitations’. We named the higher order factor as ‘locomotor disability’. This two-factor model explained over 68% of the total variance among the LD indicators. The reproduced correlation matrix indicated a good model fit with 14% non-redundant residuals with absolute values > 0.05. However, the Chi-square test indicated poor model fit (*p* < .001). The Bartlett’s test of Sphericity confirmed patterned relationships amongst the LD indicators (*p* < .001). The Kaiser-Meyer-Olkin Measure (KMO) of sampling adequacy was .94 and the individual diagonal elements in the anti-correlation matrix were > .91.

Among the retained 19 items, there was no correlation coefficient > .9 or a large number of correlation coefficients < .3. The communalities were high: between .495 and .882 with a mean of 0.684. As an evidence of convergent validity, we had all loadings above .5, except one. As an evidence of discriminant validity, we had no strong (> .3) cross loadings and the correlation between the two factors was .657. The ‘mobility activity limitations’ and ‘functional activity limitations’ sub-scales demonstrated excellent internal consistency (Cronbach’s alpha were .954 and .937, respectively).

**Conclusions:**

The 19-item LDS was found to be a reliable and valid scale to measure the latent constructs mobility activity limitations and functional activity limitations among adults with mobility impairments in outpatient and community-based settings in Bangladesh.

## Background

Disability is an evolving concept. There are several schools of thought of how to define disability and classify people who experience it [1]. The International Classification of Functioning, Disability and Health (ICF) views disability as an umbrella term for impairments, activity limitations and participation restrictions [2]. Impairments are problems relating to an individual’s body function or structure, such as paralysed legs. Activity limitations are difficulties an individual may experience in performing activities, such as toileting. Whereas, participation restrictions are difficulties an individual may experience in participating in life situations, for example participation restrictions to employment [2]. Disability represents the negative aspects of a dynamic interaction between individual health conditions and contextual (personal and environmental) factors [2]. Bangladesh is estimated to have about 17 million people aged 15 years and over with a disability [3–5]. A large proportion of them (28 to 43%) are estimated to have permanent physical impairments [6, 7]; thus, might have locomotor disability (LD)- disability relating to mobility impairment(s).

We defined LD as the resultant activity limitations and participation restrictions of the interaction between individuals’ permanent mobility impairment(s) and their personal (such as age and sex) and environmental (such as social, political and physical environmental) factors. A multi-item measurement scale is needed to measure a phenomenon like LD which cannot be measured directly, but which is believed to exist in theory [8]. We strive to achieve accuracy in measurement, but to some extent error is always introduced into the measurement process. Validity of a measurement scale focuses on the fundamental relationship between the construct/latent variable and its empirical indicators [9], therefore, validity is more of a theoretical issue than reliability [10]. Although LD is not directly measureable, it presumably has specific values under specific conditions. A LD measurement scale should be able to estimate its actual value at any given time and place. It is presumed that the underlying latent variable causes each item of a multi-item scale to score a certain value [8]. Thus, there should be a correlation between each of the item’s score and the true score of LD and, consequently, the items should also correlate with each other. We are unable to compute a correlation between a latent variable and any item used to measure it because the true score of a latent variable cannot be measured directly. However, it is possible to infer how strongly each item is correlated with its underlying latent variable by investigating how the items of a measurement scale correlate with each other [8].

There are over 350 Non-governmental Organisations (NGOs) working in Bangladesh for the betterment of disabled people, along with the Government. Many of these NGOs are involved in providing treatment and rehabilitation services to them [11]. However, Bangladesh lacks a reliable and valid scale to measure locomotor disability. Although sometimes imperfect measurement might be preferred over no measurement at all, we need to consider that if a measurement instrument is flawed, the results and any decisions based on that measurement are also accordingly flawed. Any instrument intended to measure disability among adults with mobility impairment in Bangladesh should go through a rigorous development and validation process in Bangladesh. We developed a 70-item Locomotor Disability Scale (LDS) through a qualitative study with adults with mobility impairments in Bangladesh. The scale items and scoring methods were initially developed through semi-structured interviews and later refined with cognitive interviews with adults with mobility impairments. In a previous article, we discussed further details on item generation and scoring methods of the LDS [12]. The 70-item LDS is expected to have excellent content validity and relevance to the target population considering its methods of development. However, factorial structure of the LDS was never investigated. Further, administering this long scale is time consuming and will add burden on the respondents, and therefore will increase missing responses [13]. A long instrument might also result in poor engagement of the respondents. Therefore, a shortened version of the LDS with necessary measurement properties is desirable. The objectives of this article were to develop a shorter version of the LDS and arrive at a more parsimonious conceptual understanding of the LD indicators by determining the number and nature of underlying latent variables needed to account for the pattern of correlations among the LD indicators.

## Methods

We have conducted a cross-sectional survey of 316 individuals with mobility impairments using an interviewer-administered structured questionnaire.

### Sampling and participants

We have selected our samples from the adults with mobility impairment who were accessing outpatient or community-based rehabilitation (CBR) services from the Centre for the Rehabilitation of the Paralysed (CRP), in Dhaka, Bangladesh between December 2010 and February 2011. We have recruited everybody who met our predefined inclusion criteria. The inclusion criteria for this study were adults who were aged between 18 and 65 years, had a diagnosis of any permanent or chronic mobility impairment, and were living in the community with LD at least for the past 1 month prior to the date of participation. The LDS is an interviewer administered self-reported LD measure. Therefore, individuals were excluded from this study if, in addition to their mobility impairments, they had one or more of the following problems: cognitive and perceptual problems, dementia or problems with memory, psychiatric disorders, any medical emergency.

During the data collection period we found a total of 328 eligible adults with LDs, of which 316 provided consent and completed the survey. Recommendations on appropriate sample size for studies involving factor analysis vary greatly [14]. These range from 5 participants per measured variable [15] to 10 participants per measured variable [16]. However, new evidence suggests that when at least three to four measured variables represent each common factor and communalities are high (0.70 or higher), the total sample size can be as small as 100 [17]. However, a sample size of at least 200 is recommended by Fabrigar and Wegener et al. [14]. Socio-demographic and clinical characteristics of the adults with LDs participated in this study are presented in Table 1.

**Table 1:** Characteristics of the participants

Characteristics	Participants
Age (in years)	
Mean (SD)	37.3 (13.8)
Gender	
Male	67.7%
Female	32.3%
Marital status	
Currently married	59.4%
Divorced/separated/widowed	5.7%
Never married	34.9%
Educational attainment	
Less than primary	23.4%
Primary	33.5%
Lower Secondary	14.2%
Upper Secondary or higher	28.8%
Occupation	
Unemployed	44.6%
Elementary	12.7%
Public/private service	13.0%
Business	10.4%
Student	8.2%
Housewife	11.1%
Area of residence	
Rural	66.4%
Urban	33.6%
Household monthly income	
Median	BDT 9750 (120.9 USD)
Min-Max	BDT 0.00 to 80,000.00 (0-992.2 USD)

### The locomotor disability scale (LDS)

The LDS is an interviewer-administered self-reported disability measure. It has 70 items related to mobility and functional activities. The LDS has a Likert type 5-point severity scale ranging from 0 to 4 to rate the level of difficulties an individual might encounter in performing each of the activities included in the scale. In the severity scale ‘0’ indicates no or negligible difficulty, ‘1’ indicates mild difficulty, ‘2’ indicates moderate difficulty, ‘3’ indicates severe difficulty, and ‘4’ indicates extreme difficulty [12].

### Data collection

Out of the total 316 participants, 199 were interviewed at the CRP and the remaining participants were interviewed in their community. Four interviewers and the first author were involved in data collection. The first author monitored and supervised data collection. All interviewers were university graduates and had prior experience of quantitative data collection. In addition, they received 1 week training on administering the LDS and ethical issues including obtaining informed consent. The first author and an experienced occupational therapist provided the training. The first author had past experience of supervising large-scale quantitative data collection as well as experience of working as an occupational therapist.

Before commencing data collection, the questionnaire was piloted among 12 adults with LDs at the CRP. During these interviews, the respondents were specifically asked about any difficulties they faced in answering the questions because of the language used in the questions and response categories. The feedback received was subsequently incorporated into the final version of the questionnaire.

### Administering the LDS

At the beginning of the interview, respondents were informed that they might find some activities were not relevant to them, but that for the purpose of this study, it was necessary to ask questions about all of those activities and their reply was very important. The interviewers also explained the response options/severity scale to the participants. A coloured flash card containing a description of each of the response options was displayed in front of the respondents throughout the interview. The interviewers’ task was restricted to reading out the questions and response options. They did not judge the level of difficulty the respondents were facing in performing the respective activities. It was the respondents who scored their difficulties, and the interviewers recorded their response on the form. The LDS has well defined activity items and response options which facilitated a consistent interpretation of the activity items and response options by the participants [12].

### Data analysis

#### Screening of the items and the participants

First, we checked the frequency distribution of the 70 disability indicators. We dropped items with 20% or more missing values, or 50% or more floor/ceiling effects from further analysis.

Second, we checked the engagement of the participants in completing the survey form. Response pattern of the participants with high missing responses and very low standard deviation were inspected. Suspected unengaged participants with over 10% missing responses or less than 0.25 standard deviations were dropped from any subsequent analysis. Again, we checked frequency distribution of the initially retained items with the retained participants. At this stage, any items with more than 10% missing values or 50% or more floor/ceiling effects were dropped. Missing data in the remaining items were replaced with the median.

Third, we checked correlation matrix of the retained items. We dropped items that had a large number of very low correlation coefficient (*r* < +/− 0.3) and items that had a very high correlation coefficient (*r* > +/− 0.9).

#### Exploratory factor analysis (EFA)

Factor analyses appropriate for ordered categorical variables were performed using the IBM SPSS Statistics 20. We performed EFA to empirically determine the number of constructs, or latent variables, or factors which underlie the LDS items [18]. It seeks to analyse correlations among the LDS items to explain these variables in terms of their latent variables [19, 20]. In this analysis, the latent variable is LD.

The common factor model was employed as the factor extraction model since the purpose was to understand the latent construct that accounts for the relationship among the measured variables [21] in the LDS. Oblique (promax) rotation was favoured over orthogonal rotation because oblique rotation allows factors to be correlated, and thus better represents reality and produces a simpler structure, if factors are really correlated [21].

At first, we inspected the communalities matrix. We considered deletion of any items with a coefficient bellow 0.4. Following an iterative approach, EFA was systematically re-run after the removal of each item. As a next step, cross-loading items on the pattern matrix were examined. Items with a loading of >.30 on more than one factor were considered for deletion. Further to this, we considered deletion of any items loaded on an inappropriate factor which was difficult to interpret. Again, following an iterative approach, EFA was systematically re-run after the removal of each item. We examined the Kaiser-Meyer-Olkin Measure of Sampling Adequacy (KMO) and Bartlett’s Test of Sphericity each time we ran the CFA. In addition, each time, we examined the reproduced correlation matrix for non-redundant residuals with absolute values greater than 0.05.

The decision regarding the number of factors to be retained is one of the most critical methodological decisions in factor analysis [22]. The *Kaiser-Guttman* rule and the Scree plot test were used in the EFA to decide on the number of factors to be kept [21]. The *Kaiser Guttman* rule suggests retaining factors with an eigenvalue greater than one [14, 23]. On the other hand, the Scree plot test involves visual examination of a plot of eigenvalues to identify the breakpoint at which the scree begins. Only the factors that do not belong to the scree are kept [22]. In addition, the number of factors that gives a high proportion of variance accounted for and the most interpretable solution were also considered as criteria for keeping items and factors in the LDS [21].After item reduction and determining the number of factors needed for a parsimonious conceptual understanding of the LD indicators, we computed Cronbach’s alpha to estimate the internal consistency reliability of the each sub-scale [24].

## Results

### Screening of the items and the participants

After initial descriptive analysis of the original 70 items, 26 items with more than 20% missing values and 14 items with very high (50% or more) floor/ceiling effects were dropped, leaving 30 items retained for further analysis. Then, investigation of the engagement of the participants in completing these 30 items resulted in dropping 22 suspected unengaged participants: 11 participants with more than 10% missing responses and 11 participants with .25 or less standard deviation (5 with 0 SD, 5 with 0.18 SD and 1 with 0.25 SD). Again, we checked frequency distribution with the retained 294 participants. At this stage, we dropped 1 item (“walking inside the home”) with greater than 50% floor effects and 1 item (“accessing public services”) with more than 10% missing values. This left us with 28 items. Finally, we checked the correlation matrix and dropped one item (“carrying objects”) with a large number of correlation coefficients less than 0.3 and two items (“getting out of a squatting position” and “walking around obstacles”) with correlation coefficients greater than 0.9. Eventually, we retained 25 items for EFA. Table 2 reports the frequency distribution of the 25 retained items including missing value percentages.

**Table 2:** Score distribution and missing proportion of the initially retained 25 items of the LDS

Items	Proportion					
	No difficulty	Mild difficulty	Moderate difficulty	Severe difficulty	Complete difficulty	Missing
1. Walking in the neighbourhood	21.4	32.7	12.6	4.1	29.3	.0
2. Standing up from sitting on a chair	38.8	29.9	6.5	3.7	21.1	.0
3. Maintaining a standing position	25.5	25.9	14.6	9.2	24.8	.0
4. Walking on different surfaces	4.8	32.0	23.5	8.5	31.3	.0
5. Getting into a squatting position	19.4	29.6	9.2	4.4	37.4	.0
6. Climbing up and down two flights of a stair	12.5	38.5	15.3	3.8	29.9	2.0
7. Standing up from a sitting position on the floor	20.4	31.6	17.0	5.1	25.9	.0
8. Maintaining a squatting position	23.8	27.9	9.9	5.1	33.3	.0
9. Travelling by taxi/car	46.0	21.6	11.0	8.6	12.7	1.0
10. Sitting down on the floor	29.6	29.9	11.2	5.4	23.8	.0
11. Grooming	48.3	14.3	18.4	11.9	7.1	.0
12. Dressing	44.2	24.8	17.0	7.1	6.8	.0
13. Taking a bath or shower	33.0	19.0	22.8	15.0	10.2	.0
14. Washing parts of the body	45.6	21.1	21.1	8.5	3.7	.0
15. Maintaining own health	27.9	19.4	22.8	13.9	16.0	.0
16. Toileting	29.6	24.8	24.5	11.2	9.9	.0
17. Shopping	17.4	14.5	18.8	12.7	36.6	6.1
18. Socializing	23.0	23.7	19.2	10.7	23.4	1.0
19. Attending ceremonies	18.0	21.6	16.6	8.1	35.7	3.7
20. Praying^a^	38.7	25.2	16.1	7.7	12.4	6.8
21. Reaching for overhead objects^a^	21.4	19.0	12.6	5.8	41.2	.0
22. Feeding^a^	48.0	15.6	31.6	4.8	0	.0
23. Travelling by public transports^a^	13.1	24.9	21.1	15.2	25.6	1.7
24. Getting into and out of own home^a^	41.5	29.9	12.6	3.7	12.2	.0
25. Travelling by non-motorised vehicles^a^	17.7	28.9	18.4	14.6	20.4	.0

^a^These items were dropped following EFA

### Exploratory factor analysis

First, we checked communalities matrix and following an iterative approach, EFA was systematically re-run after the removal of each item with a loading of < .4. This resulted in the consecutive deletion of the following three items: “praying”, “reaching for overhead objects” and “eating”.

As a next step to item reduction, we examined cross-loading items on the 22-item pattern matrix. Again, following an iterative approach, EFA was systematically re-run after the removal of each cross-loading (> .3) item. This resulted in the consecutive deletion of the following three items: “getting into and out of own home”, “travelling by public transports”, and “travelling by non-motorised vehicles”. Inspection of the correlation matrix of the retained 19 items revealed that there was no correlation coefficient > .9 and there are not a large number of correlation coefficients < .3. The communalities were high: between .495 and .882 with a mean of 0.684. The Bartlett’s test of Sphericity (see Table 3) confirmed that we have patterned relationships amongst the locomotor disability indicators (*p* < .001). The Kaiser-Meyer-Olkin Measure (KMO) of sampling adequacy was .94 and the individual diagonal elements in the Anti-Correlation matrix were > .91. These indicated the suitability of our data for EFA.

**Table 3:** Kaiser-Meyer-Olkin Measure of Sampling Adequacy and Bartlett’s Test of Sphericity

Kaiser-Meyer-Olkin Measure of Sampling Adequacy	.942
Bartlett’s Test of Sphericity	
Approx. Chi-Square	5807.826
Df	171
*P*	< 0.001

**Fig. 1:**
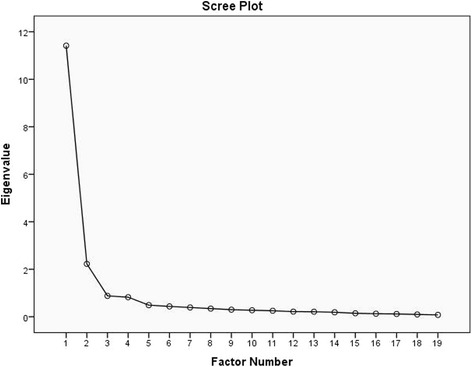
The Scree plot indicating that the data have two factors

Both the Eigen values greater than one rule and the Scree plot (Fig. 1) suggested a two-factor model. Eigen values for these two factors were 11.4 and 2.2, respectively. This two-factor model could explain 68.4% of the common variance between the 19 LD indicators. We named these two common factors as “mobility activity limitations” and “functional activity limitations”, respectively. We named the higher order factor as “locomotor disability”. The reproduced correlation matrix indicated a good model fit with 14% non-redundant residuals with absolute values greater than 0.05. However, the Chi-square test was significant (value: 904.7; df: 134; *p* < .001); which indicated a poor model fit. Table 4 presents the retained 19 items with their standardized factor loadings. As an evidence of convergent validity we had all loadings above .5, except “attending ceremonies”, which had a loading of .48. Further to this, as an evidence of discriminant validity we had no strong (> .3) cross loadings. The correlation between two factors was .657; which was another evidence of discriminant validity. The “mobility activity limitations” and “functional activity limitations” sub-scales demonstrated excellent internal consistency. Cronbach’s alpha for these subscales were .954 and .937, respectively.

**Table 4:** Rotated factor loadings

Items	Factors	
	Mobility activity limitations	Functional activity limitations
1. Walking in the neighbourhood	1.002	
2. Standing up from sitting on a chair	.996	
3. Maintaining a standing position	.913	
4. Walking on different surfaces	.891	
5. Getting into a squatting position	.841	
6. Climbing up and down two flights of a stair	.793	
7. Standing up from a sitting position on the floor	.673	
8. Maintaining a squatting position	.672	
9. Travelling by taxi/car	.630	
10. Sitting down on the floor	.582	
11. Grooming		.934
12. Dressing		.933
13. Taking a bath or shower		.911
14. Washing parts of the body		.845
15. Maintaining own health		.730
16. Toileting		.718
17. Shopping		.594
18. Socializing		.527
19. Attending ceremonies		.480

Extraction Method: Maximum Likelihood. Rotation Method: Promax with Kaiser Normalization

## Discussion

The purpose of our study was to achieve a shorter version of the LDS and to arrive a parsimonious conceptual understanding of the LD indicators retained in the LDS. In this regard, we determined the latent variables needed to account for the pattern of correlation among the set of LD indicators in the LDS. The original LDS had 70 LD indicators [12]. We investigated these LD indicators’ relevance to the population of interest, communalities and loadings to the common factors identified. Subsequently, we dropped 51 items from the original 70 items and proposed a 19-item LDS (LDS-19). The EFA analyses established a two-factor model solution, indicating that the LDS-19 measures two common factors: “mobility activity limitations” and “functional activity limitations”, and a higher order factor, “locomotor disability”. The original study which developed the contents of the LDS grouped LD indicators into two broad categories: mobility activities and occupational performance. Occupational performance activities were further divided into activities of daily living (ADL), work and leisure activities [12]. In our analysis, ADL, work and leisure activities merged to form the ‘functional activity limitations’ factor.

Determining the number of factors is a vital decision in factor analysis. Retaining too few factors may result in losing important information. On the other hand, retaining excessive number of factors makes a model complex and might result in failure to give appropriate priority to the key factor(s) [14]. Hence, establishing a balance between retaining and dropping factors constitutes the main priority in factor analysis. In our analysis Eigenvalues greater than one rule and the Scree test indicated a two–factor solution. However, we were aware that these guidelines are arbitrary [14]. The Eigenvalues greater than one rule is known to retain too many factors [22, 25]. Previous evidence suggests that the Scree test also overestimates the number of factors [22]. Hence, in retaining the two factors suggested by the Eigenvalues and the Scree test, we also considered factor loadings; model fit information and the ability of the factors to explain common variance among disability indicators, as well as evaluating theoretical explanations. These led us to proposing a two-factor model of measuring LD in adults with mobility impairment in Bangladesh. These two factors are ‘mobility activity limitations’ and ‘functional activity limitations’, with a higher order factor ‘locomotor disability’. Our proposed two-factor solution could explain over 68% of the common variance among the disability indicators. And, this is in line with the current theoretical explanation of disability. According to the international classification of functioning, disability and health, proposed by the WHO, disability is an umbrella term of impairment, activity limitations and participation restrictions [2]. Our participants are adults with mobility impairments. Their mobility impairments interacted with their personal and environmental factors and resulted in activity limitations and participation restrictions in life situations, a state we termed as ‘locomotor disability’.

Factor analysis neutralised specific variance and random error resulting in locomotor disability, which is captured by our two-factor model. This model best reflected the association between the latent factors and indicators. The implication is that “mobility activity limitations” and “functional activity limitations” serve as the latent variable that contributes to different forms of locomotor disability indicators. We favoured factor analysis over principal component analysis (PCA) because it provides more internal reliability to the scale, as it analyses only the variability in an indicator that is shared among the other indicators. In contrast, PCA analyses all variability in an indicator, including error or unique variance [26].

The indicators of the LDS-19 include mobility activities and functional activities. Some of the mobility activities, such as standing up from sitting on a chair and getting into a squatting position, rely only on physical abilities while performance in other mobility activities, such as walking in the neighbourhood and all functional activities, rely both on physical abilities and contextual factors (personal and environmental). Inclusion of these activities as locomotor disability indicators in the LDS comply with the theory that disability results from a dynamic interaction between an individual’s impairment(s) and contextual factors, such as personal attributes, attitudinal and environmental barriers [2].

The strength of the LDS-19 lies in the methods of developing the contents of the LDS. The contents of the LDS were developed by qualitative research- semi-structured interviews and cognitive interviews with adults with locomotor disabilities [12]. Developing scale contents by consulting a sample of the target population ensured that the scale has relevance to the target population and that its contents and response categories are not ambiguous to them [27, 28]. This bottom-up approach of developing the original LDS items ensured that culture specific items retained in the shortened 19-item version too. These items are getting into a squatting position, maintaining a squatting position, sitting down on the floor, standing up from a sitting position on the floor and washing parts of the body. Over 99% of the population in Bangladesh are either Muslim (89.1%) or Hindus (10%) [29]. Their religious and cultural practices require them to getting into a seated position on the floor and getting out of that position. In addition, people use low toilet, hence squatting is important. Washing parts of the body is also important, such as practicing Muslims need to perform ablution at least five times a day.

Factor analysis enabled us to determine the latent variables, “mobility activity limitations” and “functional activity limitations”, which are not apparent from direct observation of the data, but the LDS is intended to measure. Factor analysis also enabled the development of a reliable measure that can be translated into the locomotor disability score. The reliability or the internal consistency of the two-factor model was excellent as was demonstrated by the Cronbach’s alpha. The LDS-19 is an evaluative measure since it asks respondents to rate how difficult it is for them to perform the activity items [30].

An important limitation of this study was selecting respondents from only one rehabilitation centre which may have resulted in selection bias. Nevertheless, that centre is the only specialised treatment and rehabilitation centre in Bangladesh which provides inpatient, outpatient, vocational rehabilitation and community-based rehabilitation services to disabled people. It operates through a multi-disciplinary team and treats disabled people from all geographical areas of Bangladesh and all socioeconomic backgrounds [31].

## Conclusions

This study proposed a shortened version of the LDS, the LDS-19. The LDS-19 measures two latent variables: ‘mobility activity limitations’ and ‘functional activity limitations’. The higher order factor is ‘locomotor disability’. This two-factor model of measuring locomotor disability demonstrated excellent convergent and discriminant validity as was evident by factor loadings, absence of major cross-loadings, and correlations between these two factors. The scales also demonstrated excellent internal consistency reliability. In a previous study, we developed the items of the original LDS by interviewing adults with locomotor disabilities. In this study, we dropped redundant items using descriptive and factor analysis. Thus, the retained 19 items have particular importance to adults with locomotor disabilities in Bangladesh. The disability indicators of the LDS-19 include mobility activity and functional activity items. Therefore, it would be particularly suitable in evaluating rehabilitation outcomes in outpatient and community settings. We do not recommend the use of this scale in evaluating inpatient rehabilitation outcomes since the scale requires the participant to perform activities in their own community.

## Abbreviations

ADL: Activities of Daily Living
CBR: Community-based Rehabilitation
CRP: Centre for the Rehabilitation of the Paralysed
EFA: Exploratory Factor Analysis
ICF: International Classification of Functioning, Disability and Health
LD: Locomotor Disability
LDs: Locomotor Disabilities
LDS: Locomotor Disability Scale
LDS-19: 19 item Locomotor Disability Scale
NGOs: Non-governmental Organisations
